# Patient‐reported outcome measures in prostate research: a scoping review

**DOI:** 10.1111/bju.70022

**Published:** 2025-10-10

**Authors:** Grace J. Young, Eleanor I. Walsh, J. Athene Lane, Jenny L. Donovan, Marcus J. Drake, Hugo Pedder, Chris Metcalfe

**Affiliations:** ^1^ Bristol Trials Centre, Bristol Medical School University of Bristol Bristol UK; ^2^ Population Health Sciences, Bristol Medical School University of Bristol Bristol UK; ^3^ Department of Surgery and Cancer Imperial College London UK

**Keywords:** randomised controlled trials, scoping review, prostate cancer, lower urinary tract symptoms, patient‐reported outcome measures

## Abstract

**Objectives:**

To summarise how patient‐reported outcome measures (PROMs) are used in prostate research, specifically in the 10 years after the 2010 CONsolidated Standards Of Reporting Trials (CONSORT) guidelines were introduced.

**Methods:**

The search was focussed on randomised controlled trials (RCTs) reporting in the top 15 journals in oncology, urology, and medicine, during 2011–2020 and identified through PubMed®. For each article the following items were identified: the condition being treated, the intervention(s) of interest, the study design, the specific PROM(s) used, when they were included in the treatment pathway, how they were analysed, and whether methods to deal with multiplicity or missing data were considered.

**Results:**

There were 361 potentially eligible articles identified from the PubMed search, of which 121 were eligible for the full‐text review. The articles were RCTs assessing interventions for lower urinary tract symptoms (*n* = 54) or prostate cancer (*n* = 67), for which the most commonly reported PROMs were the International Prostate Symptom Score (50/54) and Functional Assessment of Cancer/Chronic Illness Therapy questionnaires (28/67), respectively. Details on the analysis and handling of PROMs were difficult to obtain; notably, 60% of articles failed to mention whether any methods had been used for dealing with multiplicity or missing data. An incidental finding was that sexually inactive men were excluded from analyses in some of the articles.

**Conclusions:**

Our scoping review highlights the need to refine the way PROMs are incorporated and analysed in prostate randomised trials, so their findings can be efficiently applied in further research and clinical practice. Adherence to the CONSORT guidelines, specifically clear reporting of the timing of PROMs, the handling of missing data, and multiplicity, should be encouraged. RCTs in prostate cancer would benefit from core outcome and measurement sets, to avoid unnecessary overlap and facilitate evidence synthesis.

**Abbreviations**
BPI: Brief Pain Inventory
CONSORT: CONsolidated Standards Of Reporting Trials
EORTC: European Organisation for Research and Treatment of Cancer
EPIC: Expanded Prostate Cancer Index
FACT/FACIT: Functional Assessment of Cancer/Chronic Illness Therapy (questionnaires)
IIEF: International Index of Erectile Function
ITT: intention to treat
LOCF: last observation carried forward
MCID: minimally clinically important difference
PCa: prostate cancer
PRISMA‐ScR: Preferred Reporting Items for Systematic Reviews and Meta‐analyses Extension for Scoping Reviews
PRO(M): patient‐reported outcome (measure)
QLQ: Quality of Life Questionnaire
RCT: randomised controlled trial

## Background

The importance of patient‐reported outcome measures (PROMs) as an endpoint in randomised controlled trials (RCTs) is increasingly recognised [1]. This is especially the case for pragmatic RCTs, which aim to measure outcomes that impact on, and are important to, study participants. A PROM is a question/questionnaire that asks trial participants to document their current state, or recent change, in general health or symptoms. The addition of PROMs, in RCTs, has gained momentum during the past few decades and is an evolving field [1, 2]. Their use has sparked changes in clinical practice by providing key information for clinician/patient shared treatment decision making [3]. Despite this new wealth of data, there is little uniformity in the way that PROMs are used. Various research groups exist to develop methods of reducing the heterogeneity of outcome measures [4] and some have applied this to prostate cancer (PCa) research [5, 6] but few have explored how these PROMs are currently being implemented and analysed.

There are several major questionnaires to assess benign and malignant conditions of the prostate. A commonly reported PROM for LUTS is the IPSS, which provides an overall score (from 0 to 35) indicating how severe a patient's symptoms are. Some questionnaires also investigate bowel and sexual symptoms, which can be affected by the prostate condition itself, or treatment side‐effects, e.g., the Expanded Prostate Cancer Index (EPIC). In advanced PCa research, the burden of the condition and treatment is sometimes better reflected through quality of life, rather than symptoms and survival [7], e.g., the European Organisation for Research and Treatment of Cancer (EORTC) Quality of Life Questionnaire (QLQ). Additionally, when looking specifically at more advanced PCa, questionnaires often cover pain and hormonal symptoms.

The way in which PROMs are captured in a clinical trial varies greatly and is often based on previous research, to aid comparison and/or confirm previous findings. First, choosing which PROMs to use, and how many, is complex and risks question overlap, leading to ethical considerations around research waste and patient burden [3]. Second, there is limited guidance on when the PROM should be incorporated into the treatment pathway. Some trialists may choose the start of treatment as the origin of measurement, allowing questionnaires to be completed at specific intervals after treatment (e.g., 6 months after the date of surgery). Others may choose randomisation as the origin and, if different from the treatment start date, this may lead to heterogeneity in the chronology of treatments and questionnaires across patients. The final obstacle is the way in which these outcomes are reported and analysed. Some triallists take the simplest cross‐sectional approach of comparing treatment arms at a specific point after randomisation or treatment commencement, adjusting for a baseline measure. Others may opt for a longitudinal approach that looks at the changing landscape of a treatment or pathway, by using PROMs at regularly spaced intervals. The latter approach has statistical advantages (e.g., the ability to cope with missing data at certain time points) but is sometimes harder to interpret [8].

There are perceived barriers when using PROM findings, from RCTs, to guide clinical decisions with patients. A survey of 396 oncologists, in 2014, highlighted a lack of understanding and interpretation of PROMs and concerns around generalisability of results [9]. Clearer reporting of PROMs may facilitate their clinical application, and the CONsolidated Standards Of Reporting Trials (CONSORT) guidelines were introduced in 1996 to aid the consistency in reporting of RCTs [10]. These were revised in 2010 [11], with an additional PROM extension in 2013 [12] and update in 2025 [13]. The CONSORT guidelines were introduced to help standardise the reporting of trial findings. They also aimed to ensure that readers are provided with all of the information they need to critically appraise, interpret and use the research, enabling systematic/scoping reviews [14].

This scoping review was designed to assess the variability, and quality in reporting, of PROMs used in prostate RCTs, focussing on the 10 years after the revised CONSORT guidelines were first introduced. It investigates which PROMs are being used in prostate research and how they are incorporated, analysed, and presented across high‐impact journals.

## Methods

This scoping review was conducted in accordance with the Preferred Reporting Items for Systematic Reviews and Meta‐analyses Extension for Scoping Reviews (PRISMA‐ScR) guidelines and checklist [15]. A protocol for this scoping review was made accessible prior to commencement, in an on‐line repository; Young GJ, Lane JA, Pedder H, et al. 2021. Bristol Research Portal, University of Bristol (unpublished work).

### Search Strategy

The aim was to identify all published RCT papers, in the medical literature, that were based on research in treatments of benign and malignant diseases of the prostate (e.g., treatments for LUTS or PCa) and included at least one PROM as an outcome. The abstract search was carried out in PubMed® and searched for keywords such as ‘RCT’, ‘patient‐reported’ and ‘prostate’; a full list of search terms is provided in Table S1: Appendix S1. Additional filters were that articles needed to be in the English language and have their full text available. As outlined in the protocol, articles were initially sourced from the top five journals from each of general medicine, urology, and oncology (15 in total). The CONSORT guidelines were updated in 2010, therefore, January 2011–December 2020 was selected for this review.

### Study Selection

Published articles were extracted from PubMed on the 12 July 2021 and managed using the software Rayyan [16]. Initially, in Stage 1, the articles were reviewed by title only. Articles were excluded as they were identified as not being reports of RCTs, e.g., responses to authors or systematic reviews. The second stage was a full abstract review, which was conducted by two researchers (G.J.Y. and E.I.W.) independently. The inclusion criteria were RCT articles that reported PROMs findings in ≥50 men, receiving treatment for conditions of the prostate. Where multiple papers had been published, analysing the same dataset, they were all included. A full list of exclusion criteria can be found in Table S2: Appendix S1. Consecutive disagreements over inclusion/exclusion of articles were discussed, with a third reviewer on hand (H.P.) in the event that no consensus could be reached; Table S3: Appendix S1. Stage 3 was the full‐text review and papers were excluded if any of the reasons for exclusion in Stage 1 or 2 had been missed. In addition, given the volume of eligible articles, papers on RCTs with smaller sample sizes (*n* = 50–100) were kept in reserve, to only be used if there were inadequate findings from the other papers.

### Data Collection

The evidence synthesis was conducted by G.J.Y., using a detailed proforma; Table S4: Appendix S1. The 24‐point checklist was developed to capture information from the included articles, about the characteristics of the trial design (e.g., number of arms, number of patients), the incorporation of PROMs (e.g., use as a primary outcome), and the analysis methods (e.g., statistical method). RCTs were assumed to be assessing for superiority, unless there were statements to suggest it was a non‐inferiority or equivalence trial. When assessing whether patients were analysed on an intention‐to‐treat (ITT) basis, words such as ‘intention’ and ‘ITT’ were searched or sentences to that effect such as ‘subjects were analysed according to the treatment they were randomised to’. The article was searched for terms such as ‘miss*’ or ‘imput*’ to determine if any methods were used to deal with missing data. It was also searched for terms such as ‘multip*’ or ‘adjust*’ to determine if any methods had been used to adjust for multiplicity; either in terms of multiple outcomes or multiple timepoints of collection. If specific items were difficult to obtain from the included article, additional searches were carried out in trial registries (e.g., clinicaltrials.gov) or published protocols. Data were collected and managed using Research Electronic Data Capture (REDCap) [17] and analysed using Stata Statistical Software, release 18.5 (Stata Corp., College Station, TX, USA) [18].

## Results

**Fig. 1:**
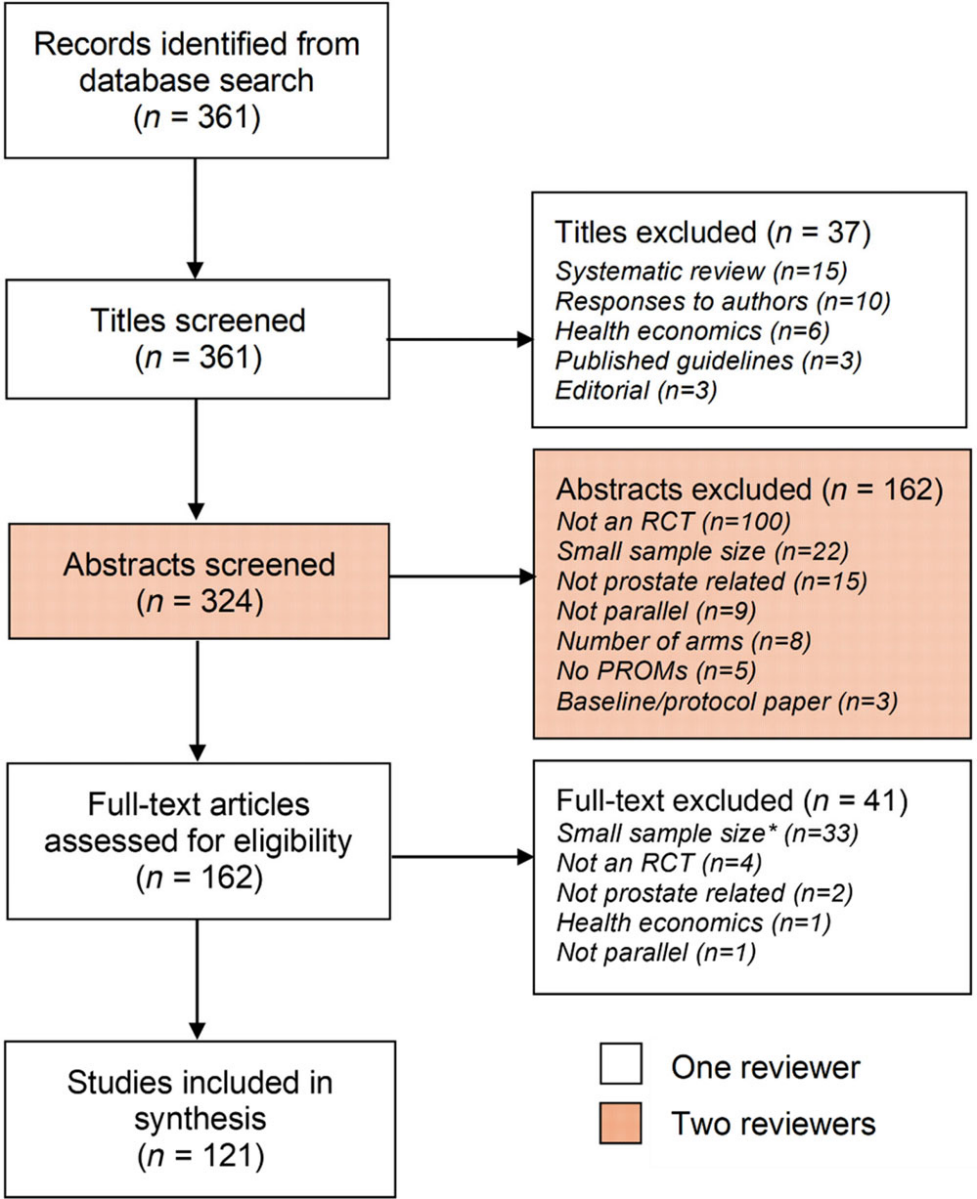
Stages of review, using a PRISMA‐ScR flow diagram. *Given the volume of eligible articles, those with sample sizes of 50–100 men were kept in reserve, to be used if conclusions could not be drawn from the other articles.

Data on 361 articles were extracted from PubMed on the 12 July 2021. After reviewing the titles, abstracts, and full texts, there were 121 full‐text articles included in the evidence synthesis: as detailed in the PRISMA‐ScR flow diagram [19] (Fig. 1). After exclusions, >60% of the articles were from three journals: *European Urology*, 26 (21%); *BJU International*, 24 (20%); and *The Journal of Urology*, 24 (20%). There were no eligible articles from three, of the initial 15, chosen journals (Table 1). Included articles are listed in Appendix S3.

**Table 1:** The RCT characteristics in the included articles (*N* = 121).

Characteristic	Category	Articles, *n* (%)
Year of publication	2011–2012	22 (18)
	2013–2014	32 (26)
	2015–2016	21 (17)
	2017–2018	23 (19)
	2019–2020	23 (19)
Journal	*Annals of Internal Medicine*	0 (0)
	*Annals of Oncology*	5 (4)
	*BJU International*	24 (20)
	*The British Medical Journal*	2 (2)
	*Cancer Discovery*	0 (0)
	*European Urology*	26 (21)
	*Journal of American Medical Association*	0 (0)
	*Journal of Clinical Oncology*	9 (7)
	*Journal of National Cancer Institute*	1 (1)
	*The Lancet*	5 (4)
	*Prostate Cancer and Prostatic Diseases*	2 (2)
	*The Journal of Urology*	24 (20)
	*The Lancet Oncology*	15 (12)
	*The New England Journal of Medicine*	7 (6)
	*The Prostate*	1 (1)
Stage of RCT	Interim (early) analysis	6 (5)
	Main outcome analysis (incl. primary)	72 (60)
	Secondary analysis	18 (15)
	Exploratory/*post hoc* analysis	16 (13)
	Subgroup analysis	2 (2)
	Longer‐term follow‐up	7 (6)
Scale of RCT	National, single site	22 (18)
	National, multisite	34 (28)
	Multinational, multisite	65 (54)
Corresponding author's location	Africa	3 (2)
	Asia	11 (9)
	Australasia	6 (5)
	Europe	55 (45)
	North America	46 (38)
Randomisation	Individual	121 (100)
	Cluster	0 (0)
Total sample size	101–300	49 (41)
	301–500	14 (12)
	≥501	58 (48)
Number of arms	2	95
	3	20
	4	6
Type of trial	Superiority	92 (76)
	Non‐inferiority	26 (21)
	Equivalence	3 (2)
Eligible ages	All adult men (≥18 years)	62 (51)
	Minimum/maximum age restriction	59 (49)
Condition under investigation	LUTS	54 (45)
	PCa	67 (55)
Primary aim of interventions	Improve symptoms	64 (53)
	Improve survival	32 (26)
	Improve progression‐free survival	15 (12)
	Other	10 (8)
What type of outcome is the primary outcome?	Clinical	63 (52)
	PROM	45 (37)
	Both (co‐primary/composite)	13 (11)

### Trial Details

More than half of the articles were multinational RCTs, with sites in more than one country (54%), and the majority were reporting primary outcome results (60%). Over 80% of corresponding authors were based in Europe or North America (Table 1). Although there were 121 articles, they were spread across 102 different RCTs as 15 of the trials had two or more eligible articles each. For example, the NEPTUNE trial (ClinicalTrials.gov identifier: NCT01018511) presented their primary [20], longer‐term follow‐up [21], and exploratory [22] analyses in three separate eligible articles during the review period (2011–2020).

Patient‐reported outcome measures were utilised in the primary outcome for nearly half of the articles, with 45 (37%) utilising solely a PROM and 13 (11%) utilising it in a co‐primary/composite primary outcome (Table 1). Of those that solely used a PROM as their primary outcome, 32 (71%) listed a minimally clinically important difference (MCID) for it but only 13 provided an accompanying reference. In those that provided a reference, 11 referenced previous trial papers as justification for their MCID, while two referenced supporting research for adopting an MCID of 0.5 SDs; as is common practice [23].

There were 29 (23%) articles reporting findings from a non‐inferiority or equivalence design and 92 (77%) from a superiority design (assumed if not explicitly stated otherwise). The main conditions of interest were PCa (*n* = 67) or general LUTS (*n* = 52). There were two condition‐specific articles on chronic prostatitis pelvic pain and erectile dysfunction, which were included as LUTS symptoms for the purposes of this review. Just over half of the articles, 64/121 (53%), reported findings from RCTs where the main aim was to improve symptoms; 49/54 (91%) of the LUTS articles and 15/67 (22%) from PCa articles. There were 32 (26%) articles presenting RCTs aiming to improve survival and 15 (12%) to improve progression‐free survival; all of which were PCa articles.

For the LUTS articles (*n* = 54); 19 used a surgical/laser intervention, 30 used a pharmaceutical or hormonal treatment, three used an ‘other’ treatment, and two used a combination of approaches (Table 2). For the PCa articles (*n* = 67); 11 used a surgical/laser intervention, 28 used a pharmaceutical or hormonal treatment, six used radiotherapy, three used chemotherapy, two used a lifestyle change (i.e., diet or exercise), one used a web‐based tool, and 16 used a combination. Both of these factors (condition and intervention) influenced the trial design. For example, LUTS articles were more likely to blind participants to their treatment allocation than PCa trials (67% vs 36%) and articles that utilised a radical treatment option (such as surgery or radiotherapy) were less likely to blind participants. The median length of the study was much longer for PCa articles than LUTS articles (24 vs 6 months) and this was reflected in the median number of PROM assessment points (six vs three). For the LUTS articles the median was three measurements across all of the interventions. However, this differed across the PCa articles, with lifestyle changes having the lowest number (two) and chemotherapy having the highest number (16), owing to measurements being taken at each treatment cycle in chemotherapy.

**Table 2:** Study design and incorporation of PROMs, according to condition (LUTS or PCa) and interventions.

Type of intervention*	Number of articles	ITT^†^, *n/N* (%)	Blinded, *n/N* (%)	Length of study, months, median (IQR)	PROMs, *n*, median (IQR)^‡^	PROM origin^§^ = start of treatment, *n/N* (%)	PROM^¶^ primary outcome, *n/N* (%)
**All LUTS interventions**	54	40/54 (74)	36/54 (67)	6.0 (2.8–18.0)	3 (3–5)	35/54 (65)	42/54 (78)
Surgery/laser	19	16/19 (84)	7/19 (37)	12.0 (6.0–24.3)	3 (3–4)	13/19 (68)	14/19 (74)
Pharmaceutical/hormonal	30	21/30 (70)	26/30 (87)	3.0 (2.8–12.0)	3 (2–5)	18/30 (60)	24/30 (80)
Other	3	3/3 (100)	2/3 (67)	12.0 (5.6–18.0)	3 (3–5)	2/3 (67)	3/3 (100)
Mixed	2	0/2 (0)	1/2 (50)	4.2 (2.8–5.6)	3 (3–3)	2/2 (100)	1/2 (50)
**All PCa interventions**	67	58/67 (87)	24/67 (36)	24.0 (12.0–61.8)	6 (4–10)	26/67 (39)	16/67 (24)
Surgery/Laser	11	9/11 (82)	3/11 (27)	15.0 (12.0–146.0)	4 (3–5)	5/11 (45)	7/11 (64)
Pharmaceutical/hormonal	28	25/28 (89)	17/28 (61)	24.0 (15.0–42.0)	7 (5–15)	10/28 (36)	3/28 (11)
Radiotherapy	6	3/6 (50)	1/6 (17)	17.1 (3.0–60.8)	6 (3–7)	3/6 (50)	1/6 (17)
Chemotherapy	3	3/3 (100)	0/3 (0)	48.0 (24.3–50.0)	16 (10–22)	1/3 (33)	0/3 (0)
Lifestyle change	2	2/2 (100)	0/2 (0)	9.0 (6.0–12.0)	2 (1–2)	2/2 (100)	1/2 (50)
Other	1	1/1 (100)	0/1 (0)	24.0 (N/A)	4 (N/A)	0/1 (0)	1/1 (100)
Mixed	16	15/16 (94)	3/16 (19)	30.0 (12.0–79.1)	5 (4–8)	5/16 (31)	3/16 (19)

Blinded, refers to the patient being blinded to their study allocation; IQR, interquartile range (25th percentile, 75th percentile); ITT, analysed according to the ‘Intention to Treat’ principle; N/A, not applicable.

* Placebo/no intervention/surveillance are not considered here, as they were considered comparator arms.

^†^ Where this was not explicitly mentioned in the manuscript (28/121) it was assumed that the analyses were by ITT.

^‡^ The number of times a single PROM was measured, e.g., twice if measured at 6 and 12 months. Unclear in three PCa articles.

^§^ Other options included randomisation, removal of catheter, or end of treatment.

^¶^ Either on its own or as part of a composite outcome or co‐primary.

The number of articles analysing treatments on an ITT basis was 98 (81%), and similar across the interventions (Table 2). However, only 70 articles explicitly stated using ITT and it was inferred for 28 articles where there was no statement around the use of ITT or alternative method. Of those RCT articles that were not analysed on an ITT basis (*n* = 23), the majority quoted ‘per protocol’ and 11 were from non‐inferiority/equivalence trials; where it has been argued that per protocol analyses may be more appropriate [24]. There was a slightly greater proportion of PCa articles using ITT than LUTS articles (87% vs 74%), yet only half of the radiotherapy trials (three of six) used ITT.

### The PROM Details

**Fig. 2:**
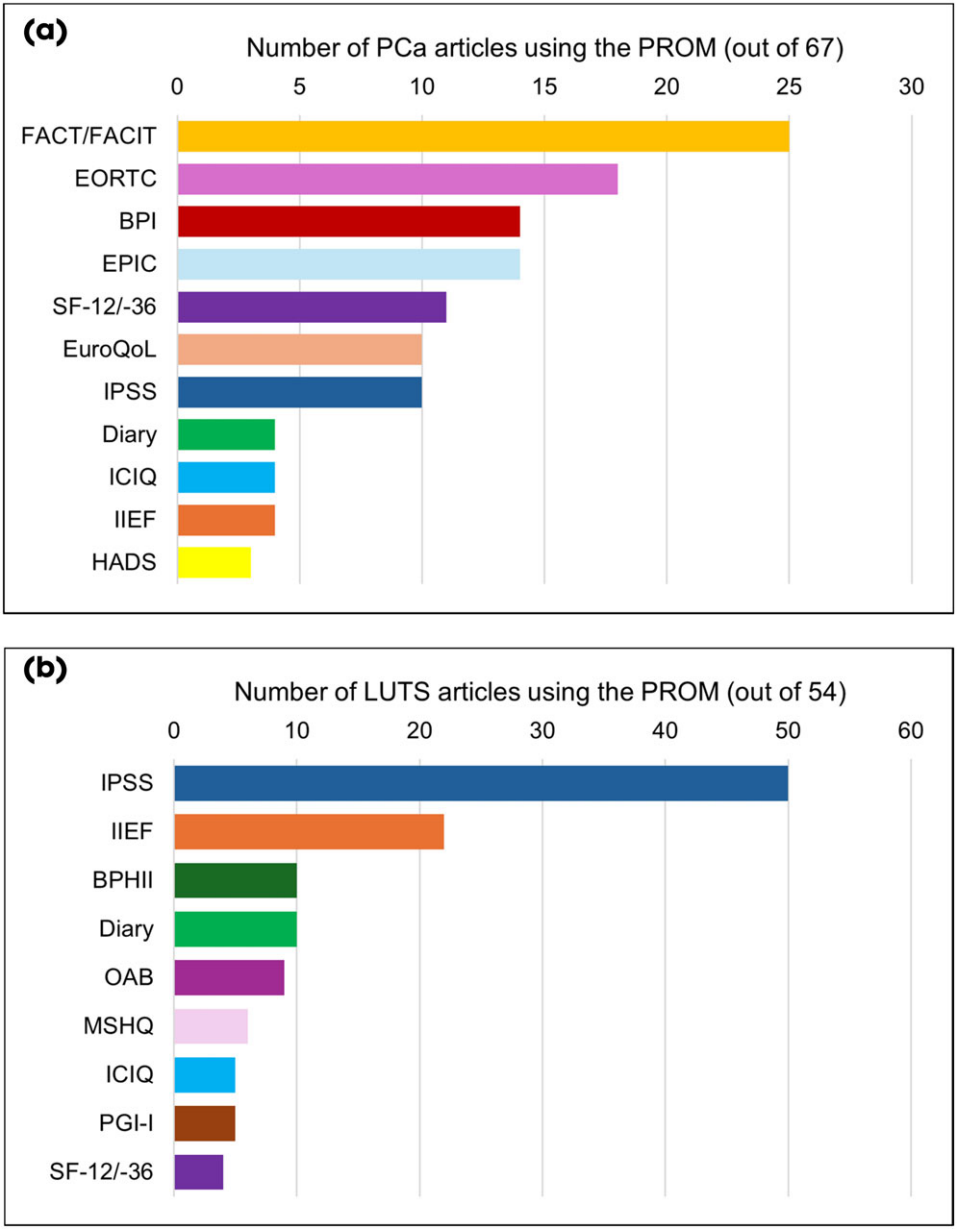
Popularity of PROMs based on the number of articles that used them across (**a**) 67 PCa articles* and (**b**) 54 LUTS articles*. *Excludes measures used in two or fewer articles, for more details see Appendix S2. BPHII, Benign Prostatic Hyperplasia Impact Index; Diary, any bladder diary (e.g., Total Urgency and Frequency Score); HADS, Hospital Anxiety and Depression Scale; ICIQ, The International Consultation on Incontinence Questionnaire; MSHQ, Male Sexual Health Questionnaire; OAB, Overactive Bladder Assessment Tool; PGI‐I, Patient Global Impression of Improvement; SF‐12/−36, 12‐ or 36‐Item Short Form Survey.

The PROMs used in the 67 PCa articles focussed heavily on health‐related quality of life. The most commonly reported PROM in the PCa articles were the Functional Assessment of Cancer/Chronic Illness Therapy (FACT/FACIT) questionnaires, with 37% of PCa articles using one of their questionnaires (Fig. 2a, Table S5a,b: Appendix S2). This was closely followed by the EORTC QLQ, which was used by 27% of articles. The third most common questionnaire focussed on the symptom of pain; the Brief Pain Inventory (BPI), with using 21% it.

The PROMs used in the 54 LUTS articles largely focussed on urinary and sexual symptoms (Fig. 2b). The most commonly reported PROM in the general LUTS articles was the IPSS, which focuses on urinary symptoms, with 93% of articles using it. The International Index of Erectile Function (IIEF) was the second most frequently used PROM, which focuses on sexual symptoms, with 41% using it. An incidental finding, when conducting this review, was the way in which sexual PROMs were handled. In some instances (at least eight articles), articles explicitly stated that they excluded individuals that were not sexually active, e.g., ‘Analyses of IIEF scores were performed using results from these patients who were also sexually active with a female partner’ [25].

The use of each PROM was relatively equal across continents, although, European questionnaires such as the EORTC QLQ and EuroQol were most commonly found in articles with corresponding authors based in Europe. Where articles reported using only one PROM, in isolation (*n* = 41), the most commonly used were the IPSS (29%), EORTC QLQ (24%) and FACT/FACIT (10%) questionnaires. Where more than one PROM was reported, the most common combinations were IPSS and IIEF (for LUTS) and FACT/FACIT and BPI (for PCa); Table S6a,b: Appendix S2.

### Analysis Details

The PROMs were analysed in various ways across the articles in both primary/secondary analyses and as sensitivity analyses. These included analysing them cross sectionally (*n* = 88), longitudinally as repeated measures (*n* = 37), and in time to event analyses (*n* = 29), e.g., time to deterioration of symptoms (≥4 points on the IPSS). In 66 articles, only cross‐sectional analyses were used, despite the majority of these articles (77%) having taken more than two measures of the PRO after baseline and 27% taking five or more measures.

The cross‐sectional analyses covered the typical parametric and non‐parametric analysis methods expected in RCTs, with the majority undertaking a *t*‐test, regression or AN(C)OVA model (Table 3). The most common longitudinal analysis was the mixed‐effects model for repeated measures, which was described in various ways including mixed‐effects model, two level random effects model, mixed‐effects model with time interaction, and repeated measures ANOVA. Graphs were used in 89 articles, to present the PROM results, sometimes using multiple types of graphs. The most common graph used was a line graph, generally presenting means and 95% CIs over time (*n* = 58; Table 3).

**Table 3:** The number of articles* using each statistical analysis test or graphical presentation, according to the number of times the PROs were measured.

Statistical analysis method or graphical presentation used	Number of times the PRO was measured after baseline					
	1	2	3	4	5	6+
**Cross‐sectional analysis methods**						
Descriptives only, no test	0	0	0	0	2	0
Pearson's chi squared/Fisher's exact test	0	3	5	6	3	6
Cochran–Mantel–Haenszel	0	0	1	3	0	4
Spearman rank correlation coefficient	0	0	1	0	0	0
*t*‐test/ANOVA	5	4	13	6	6	4
Mann–Whitney *U*/Wilcoxon rank sum/Kruskal–Wallis tests	2	2	6	7	2	5
Wilcoxon signed rank/Sign tests	1	1	0	1	0	0
Cohen's *D*	0	0	0	1	0	0
Regression models/ANCOVA^†^	0	5	14	3	4	8
Relative risk reduction, at a specific time	0	0	0	0	0	1
**Longitudinal analysis methods**						
Mixed‐effects model for repeated measures	0	2	3	6	4	16
Generalised estimating equations	0	0	0	2	1	1
Area under the curve	0	0	0	0	0	3
Pattern mixture modelling	0	0	0	1	1	0
Wei Lachin	0	0	0	1	0	0
**Time‐to‐event analysis methods**						
Kaplan–Meier and/or log rank^‡^	0	0	3	2	3	14
Cox proportional hazards	0	0	0	1	1	13
**Graphical presentations**						
Bar chart	2	4	7	8	4	5
Box plot	0	0	4	1	0	1
Line graph	0	5	16	8	8	21
Forest plot^†^	1	1	0	0	0	1
Kaplan–Meier curve	0	0	2	2	2	10
Other graph	0	0	0	1	1	1

* The numbers in each cell refer to the number of articles in the cross tabulation. Values within columns are not mutually exclusive, some articles used more than one test or graphic.

^†^ Also used by one additional article where the number of PROMs was unclear.

^‡^ Also used by two additional articles where the number of PROMs was unclear.

Multiplicity was adjusted for in 26 (21%) articles, it was explicitly stated that it was not used in 23 (19%) articles and was not mentioned in 72 (60%) articles. This was equally distributed across the journals, rather than specific to a select few, and remained consistent over the 10‐year period. Of those that used it, the most common methods included *P* value adjustment techniques, e.g., Bonferroni [26] (*n* = 19), a hierarchical approach, e.g., fixed‐sequence strategy (*n* = 4) or a step up/down approach, e.g., Benjamini–Hochberg [27] (*n* = 3).

There were similar findings when looking at imputation methods for missing PROM data; 30 (25%) articles utilised an imputation method in either a primary/secondary analysis (*n* = 21) or sensitivity analysis (*n* = 9), 18 (15%) explicitly stated that they had not used it and 73 (60%) did not mention it. Of those that used it, the most common methods were LOCF (*n* = 16), pattern mixture modelling (*n* = 6) and multiple imputation (*n* = 3).

## Discussion

This review provides an insight into how PROMs are incorporated and analysed in contemporary RCTs evaluating treatments of prostate conditions. In RCTs concerned with LUTS, the focus was on urinary (e.g., IPSS) and sexual (e.g., IIEF) symptoms, often in conjunction. There was much more variation in the PROMs used in PCa trials, which included quality of life (e.g., FACT/FACIT) and pain (e.g., BPI) as well as urinary, sexual, and bowel symptoms. While the majority of PROMs used in conjunction covered different symptoms, in some cases it could be argued that there was unnecessary overlap of questions, e.g., using the IIEF and Male Sexual Health Questionnaire in conjunction. This overlap is potentially a waste of research resources and participant time [3]. The problems and issues arising from inconsistency in outcome selection and reporting in PCa RCTs has been highlighted before [28, 29] and there are research groups that have attempted to develop a core outcome set for PCa. For example, Martin et al. (2015) [30] suggest that the EPIC‐26 be used to cover all symptoms in localised PCa. More recent publications by Ratti et al. (2022) [6] and Beyer et al. (2022) [31] suggest using the EORTC QLQ, and whether this will be internationally accepted remains to be seen.

The vast majority of articles presented the aggregated summary scores from their chosen PROM(s), rather than the individual items/scales. This made the articles easier to compare and collate, leading to better interpretability. However, when assessing certain conditions, an overall summary score may not be appropriate. For example, one article stated that ‘The individual scales were preferred over the summary measures for this analysis because each represents a single health area; we wanted to assess potential changes due to treatment over time in each health area without having a negative change in one area obscured by a positive change in another’ [32]. However, where relevant, accounting for multiplicity would need to be considered.

All bar one of the journals included in the final text review recommend or require the use of the CONSORT checklist. Non‐adherence to the checklist, despite journals’ positive endorsement, has been reported previously [33, 34]. Multiplicity was not mentioned in 60% of articles, despite being in the 2010 checklist (#20). The handling of missing data was not mentioned in 60% of articles, and these percentages remained constant, rather than improved, over the 10‐year inclusion period. While it can be assumed that missing data were ignored in these manuscripts, it would be better for articles to make explicit statements, rather than to just assume it is ‘not applicable’ and not leading to biased estimates. This lack of reporting has also been highlighted in breast cancer RCTs [35]. The inclusion of a missing data statement was not in the CONSORT 2010 checklist but was in the CONSORT extension for PROs 2013 checklist (#P12a) and has now been included in 2025 checklist (#21c) [11, 12, 13]. The most common method for dealing with missing data was LOCF (*n* = 16), which is surprising given this approach is well known to make strong assumptions about the missing data with additional bias resulting if they are not met [8, 36, 37].

Although the majority of RCTs quoted an MCID in their methods, when utilising a PROM as their primary outcome, there were still 29% that did not provide one and 42% that provided one but without justification, e.g., a supporting reference. While an MCID is not an official requirement on the CONSORT 2025 checklist, #16a states: ‘How sample size was determined, including all assumptions supporting the sample size calculation’. Therefore, it is best practice to provide an MCID, with justification, to adequately satisfy this item on the checklist.

Any exclusions from an analysis set should be detailed and justified, as required by #16 and #21b/26 for the 2010 and 2025 checklists, respectively. There were at least eight articles that excluded sexually inactive men from analyses. One argument for doing this is that the inclusion of men who do not attempt sexual intercourse may lead to an underestimate of erectile function [38].The counter argument to this is that their removal leads to a biased result, given that sexual inactivity becomes an intercurrent event in this situation, and symptoms may have led to the non‐attempt at sexual intercourse; i.e., the data are not ‘missing at random’. Our suggestion would be to include an additional question, asking the reason for sexual inactivity, e.g., ‘due to urinary symptoms’, ‘due to treatment side effects’ or ‘other reason’. Even with this question, we recommend that all men be included in the main trial's ITT analyses and only excluded in sensitivity analyses that explore the missing data assumptions [8]. More questionnaire guidance is needed to ensure triallists correctly identify which participants should be included in analyses. This also applies to other groups of individuals such as those with an indwelling catheter completing a urinary symptom questionnaire or those with a colostomy bag completing a bowel symptom questionnaire.

### Strengths and Limitations

There were various decisions made, when designing this review, which resulted in strengths and limitations. Our eligibility criteria excluded articles that were not in the English language, which ensured that all articles were immediately accessible to the research team. However, this may have limited our generalisability of PROMs use across the world as 83% of articles were from Europe or North America. We also wanted to cover a broad range of benign and malignant prostate conditions, which allowed us to cast the net wide and report on a larger variation of trials. However, this meant we were unable to identify trends within specific conditions. For example, all severities of PCa were included in this review, from localised to metastatic hormone resistant, which may have contributed to the wide selection of PROMs found. Articles on RCTs with <100 participants (*n* = 33) were not included in the full‐text review as these were likely to be underpowered, and the methods/reporting were less likely to be reliable.

### Future Work

There were various items we set out to synthesise as part of this review that we were unable to gather enough information on. We had hoped to assess whether PROMs were static (i.e., at that moment) or transitional (i.e., change since treatment) but reports were often insufficiently clear to make this distinction. We had also planned to assess how adjuvant treatments and death were accounted for in longitudinal analyses of PROMs but, unfortunately, this was not clear enough to obtain. As a general rule, articles rarely mentioned adjuvant therapies. However, we were aware of one article that explicitly stated how they handled it: ‘For ITT analysis, any subject that underwent additional BPH therapy (procedure or medication) was treated as a treatment failure and was assigned a zero reduction from baseline [in IPSS]’ [39]. While research groups continue to discuss how intercurrent events can be handled, it was evident that there were no mainstream methods for dealing with them, in the eligible articles [40, 41].

There were various article attributes that were not easily obtainable: (i) the origin of PROM measurements (e.g., randomisation or treatment commencement), (ii) the blinding status of participants completing the questionnaire, and (iii) the number of times the PROM was collected after baseline. These items were often sought from trial registries, despite these being a CONSORT methods requirement (#6a) and included in a participant flow as per the PRO extension 2013 (#13a). The review has highlighted a need for authors to be encouraged to meaningfully adhere to the CONSORT guidelines, with specific focus on the updated 2025 guidelines and PRO extension guidelines. Journals and their editors/reviewers also have a part to play in ensuring the guidelines are followed. Statistical reporting guidelines have been provided by four urology journals, since 2019 [42]. The group Setting International Standards in analysing Patient‐Reported Outcomes and Quality of Life Endpoints (SISAQOL) have also published some international standards for analysing PROMs in RCTs and there are ongoing developments of these recommendations [43, 44]. The hope is that they will provide more consistency in reporting of PROM data in oncology, going forwards. Once these guidelines are firmly in place, and endorsed by journals, a revisit of the research questions in this scoping review would be worthwhile.

## Conclusions

This synthesis of the use of PROMs in prostate‐related RCTs has highlighted several areas requiring improvement. First, the heterogeneity and unnecessary overlap in the use of PROMs, in PCa, would benefit from an internationally acceptable core outcome set. Recent systematic reviews and consensus groups have recommended the EORTC QLQ as a suitable PROM to cover all symptoms in PCa and it remains to be seen whether this approach will be adopted by new studies going forwards. Second, additional justification is needed for important decisions such as MCIDs and exclusion of participants from analyses (e.g., sexually inactive men), which can impact on overall trial findings and their interpretation. Most importantly, there is a clear message that adherence to the CONSORT guidelines needs to be more closely monitored, especially with regards to dealing with multiplicity or missing data. More consistency in reporting would lead to more efficient conversion of research into clinical practice and, in turn, better patient outcomes.

## Disclosure of Interests

The authors declare no conflicts of interest in conducting this review.

## Funding

Grace J. Young, J. Athene Lane and Chris Metcalfe receive support from the UK National Institute for Health Research (NIHR), as members of the Bristol Trials Centre. Grace J. Young received NIHR funding, as part of a predoctoral fellowship, while undertaking this work. Jenny L. Donovan is an Emeritus NIHR Senior Investigator. Marcus J. Drake reports being on associated advisory boards; has received grants, personal fees, and non‐financial support from Astellas Pharma and Viatris; and has also received personal fees from Pfizer. Hugo Pedder was supported by funding from the National Institute of Health and Care Excellence's Guidelines Technical Support Unit and Bristol's National Institute for Health and Care Research Technology Assessment Group. The views and opinions expressed are those of the authors and do not necessarily reflect those of the NIHR, the NHS, or the Department of Health and Social Care.

## Supporting information


**Appendix S1.** Scoping review screening.

**Appendix S2.** Patient reported outcome measures.

**Appendix S3.** Included articles.

**Appendix S4.** The PRISMA‐ScR checklist.
