# Supplementary material for: Patient‐reported outcome measures in prostate research: a scoping review
Source: BJU Int. 2025 Oct 10;137(2):241–50. doi: 10.1111/bju.70022 (PMC12789852; doi:10.1111/bju.70022)
Supplement: Supplementary file 1 — Appendix S1. Scoping review screening. [file BJU-137-241-s002.docx]

**Appendix 1. Scoping review screening**

**Table S1.** The number of articles returned by PubMed® (accessed 12/07/21)

| **Search terms** | **Additional filters**~ | **Years** | **Papers** |
| --- | --- | --- | --- |
| ((prostat*[Title/Abstract]) OR ((lower urinary tract[Title/Abstract]) AND (men[Title/Abstract]))) AND ((patient-reported[Title/Abstract]) OR (patient reported[Title/Abstract]) OR (quality of life[Title/Abstract]) OR (self-report*[Title/Abstract]) OR (symptom score[Title/Abstract])) AND ((random*[Title]) OR (trial[Title])) NOT (review[Publication Type]) | Language: English  Text availability: Full text | 1984-2000 | 151 |
|  |  | 2001-2010 | 276 |
|  |  | 2011-2020 | 361 |

* PubMed.gov®, Courtesy of the U.S. National Library of Medicine

~In addition the search was restricted to the top 5 journals in each subject of medicine, oncology and urology (15 in total). For more details, please refer to the protocol: Young GJ, Lane JA, Pedder H, et al 2021. Bristol Research Portal, University of Bristol (unpublished work).

**Table S2.** Abstract exclusion criteria proforma

| **Inclusion criteria** | **Exclusion criteria; examples** | **Exclusion code** |
| --- | --- | --- |
| RCT | Non-RCT, e.g. observational study | Non-RCT |
|  | *Pooled results from multiple studies^a^* | Non-RCT |
| Prostate only (males only) | Conditions outside of the prostate e.g. rectal, testicular or a mix of cancer patients | Non-prostate |
| $\geq$50 patients | <50 patients | Sample size |
| Parallel group design | Non-parallel, e.g. cross-over design | Non-parallel |
|  | *Dose escalation^a^* | Non-parallel |
| 2-4 arms | 1 or 5+ arms | Arms |
| Clinical main trial results | Health Economic results/*QALY modelling^a^* | Health Economic |
|  | Baseline findings | Baseline |
|  | Protocol paper | Protocol |
|  | Statistical Analysis Plan | SAP |
| RCT main manuscript | Commentary | Wrong manuscript type |
|  | Review/Systematic Review/Meta-analysis | Wrong manuscript type |
|  | Updated treatment guidelines | Wrong manuscript type |
|  | Editorial | Wrong manuscript type |
| PROM findings | No PROMs mentioned (however, this could be hidden by using the phrases “quality of life”). Exclude trials that only used PROMs for randomisation (baseline) as we want at least one measure after treatment. | No PROM |

^a^Specific exclusion criterion (in italics) were identified and included during the abstract review period

**Table S3. Abstract review agreement**

|  | | Reviewer 1 (GY) | | |
| --- | --- | --- | --- | --- |
|  |  | Included | Excluded | Total |
| Reviewer 2 (EW) | Included | 142 | 17 (4) | 159 |
|  | Excluded | 22 (16) | 143 | 165 |
|  | Total | 164 | 160 | 324 |

Numbers in brackets indicate the number finally included, after discussion between the reviewers

**Table S4.** Full text review extraction items

| **Criterion** | **Response** |
| --- | --- |
| ***PART 1: Trial details*** | |
| 1a. Manuscript number (ID):  1b. Trial name (if applicable):  1c. Trial number (s): | ____________  ____________  ____________ |
| 2. Manuscript type | [ ] Interim analyses  [ ] Main outcome analyses  [ ] Exploratory analyses  [ ] Longer term follow up analyses |
| 3a. Scale  3b. Country of corresp. author | [ ] National, single site  [ ] National, multi-site  [ ] Multinational, multi-site |
| 4. Randomisation  *Please tick one* | [ ] Cluster  [ ] Individual  [ ] Other: |
| 5a. Population age  *Please tick one*  5b. *If* restricted, provide details | [ ] All ages (≥18)  [ ] Restricted to specific age range |
| 6. Sample  *Please tick one* | [ ] Small: 50-100  [ ] Medium: 101-300  [ ] Large: 301-500  [ ] Very large: 501+ |
| 7a. Condition under investigation  *Multiple choice*  7b. *If* they have a prostate cancer diagnosis, how severe: | [ ] Urinary retention  [ ] Chronic prostatitis (pelvic pain syndrome)  [ ] BPH/BPE/BOO or equivalent  [ ] Prostate cancer  [ ] Other:  [ ] Advanced  [ ] Localised  [ ] Other: |
| 8. Number of arms |  |
| 9. Type of trial *(If not mentioned, assume superiority)* | [ ] Superiority  [ ] Non-inferiority  [ ] Equivalence |
| 10a. Interventions included  *Multiple choice* | [ ] No intervention (e.g. placebo or sham treatment)  [ ] Lifestyle changes (e.g. fluid intake, dietary or exercise)  [ ] Surveillance (e.g. PSA monitoring, watchful waiting)  [ ] Herbal remedy (e.g. nettle extracts)  [ ] Pharmaceutical (e.g. tamsulosin)  [ ] Diagnostic testing (e.g. UDS, biopsy)  [ ] Surgery (e.g. TURP)  [ ] Microwaves/Heat (e.g. TUNA, TUMT)  [ ] Laser therapy (e.g. HoLEP)  [ ] Radiotherapy  [ ] Artery Embolization  [ ] Other: |
| 10b. Aim of intervention | [ ] Improve symptoms  [ ] Improve survival  [ ] Other: |
| 11. Length of study or longest follow-up presented | ____________ days |
| 12. Point of randomisation  *Please tick one* | [ ] Before treatment (e.g. consultation for condition)  [ ] Commencement of treatment (e.g. surgery)  [ ] Other: |
| 13. Was the patient blinded to their allocation? | [ ] Yes  [ ] No  [ ] Unclear/not mentioned |
| 14a. What type of outcome is the primary outcome? | [ ] Clinical  [ ] PROM  [ ] Both (co-primary) |
| 14bi. *If* PROM, was an MCID/delta quoted in the sample size calculation? | [ ] Yes, with reference  [ ] Yes but without a reference  [ ] No |
| ***PART 2: PROM details*** | |
| 15. Which PROMs were collected |  |
| 16. Was the analysis intention to treat? | [ ] Yes  [ ] No  [ ] Unclear/not mentioned |
| 17a. Was a baseline measure of the key PROMs taken? | [ ] Yes  [ ] No  [ ] Unclear/not mentioned |
| 17b. Number of times measured (not incl. baseline) |  |
| 18. Origin of measurements | [ ] Randomisation  [ ] Intervention start  [ ] Other: |
| 19. Was there any imputation for missing data | [ ] Yes, in a primary/secondary analysis  [ ] Yes, in a sensitivity analysis  [ ] No  [ ] Unclear/not mentioned |
| 20. Was there any accounting for multiplicity (e.g. Bonferroni) | [ ] Yes  [ ] No  [ ] Unclear/not mentioned |
| 21. How were the PROMs included in the analysis? | [ ] Single measure  [ ] Repeated measures |
| 21. Name all statistical analysis methods used for the PROMs (e.g. linear regression) |  |
| 22a. Were any graphics used (in the main paper) to present the PROMs findings?  22b. *If* yes, name them | [ ] Yes  [ ] No |
| Notes: | |
