# Supplementary material for: Patient‐reported outcome measures in prostate research: a scoping review
Source: BJU Int. 2025 Oct 10;137(2):241–50. doi: 10.1111/bju.70022 (PMC12789852; doi:10.1111/bju.70022)
Supplement: Supplementary file 2 — Appendix S2. Patient reported outcome measures. [file BJU-137-241-s003.docx]

**Appendix 2. Patient Reported Outcome Measures**

**Table S5a.** Details of the patient reported outcome measures (PROMs) used in articles concerning prostate cancer (144 PROMs across 67 articles)

| **PROM name** | **Long name** | | **Main symptoms of interest** | **n (%) of articles using it** |
| --- | --- | --- | --- | --- |
| ***Articles concerning Prostate Cancer*** | | | | |
| FACT/FACIT measures (e.g. FACT-P) | | Functional Assessment of Cancer Therapy/Chronic Illness Therapy | QoL | 25 (37%) |
| EORTC (e.g. _C30/_PR25) | | European Organisation for Research and Treatment of Cancer | QoL | 18 (27%) |
| BPI | | Brief Pain Inventory | Pain | 14 (21%) |
| EPIC | | Expanded Prostate Cancer Index/ UCLA Prostate Cancer Index | Urinary, Sexual & Bowel | 14 (21%) |
| EuroQol (e.g. EQ-5D-5L) | | EuroQol Research Foundation | QoL | 10 (15%) |
| IPSS | | International Prostate Symptom Score | Urinary/QoL | 10 (15%) |
| SF-36/SF-12 | | 36 Item/12 Item Short Form Survey | QoL | 9 (13%) |
| Diary | | Bladder diary (e.g. TUFS) | Urinary | 4 (6%) |
| ICIQ (e.g. ICIQ-MLUTS or ICS) | | The International Consultation on Incontinence Questionnaire | Urinary/Sexual/QoL/Satisfaction with treatment | 4 (6%) |
| IIEF | | The International Index of Erectile Function | Sexual | 4 (6%) |
| HADS | | Hospital Anxiety and Depression Scale | Anxiety/Depression | 3 (5%) |
| BFI | | Brief Fatigue Inventory | Fatigue | 2 (3%) |
| IES-R | | Revised Impact of Event Scale | Stress Disorders | 2 (3%) |
| VAS scale | | Visual Analogue Scale - Urinary | Urinary | 2 (3%) |
| Viazey | | Vaizey Fecal Incontinence Score | Bowel | 2 (3%) |
| AMS | | Ageing Males Symptoms Scale | QoL | 1 (1%) |
| AQoL | | Assessment of Quality of Life | QoL | 1 (1%) |
| BPHII | | Benign Prostatic Hyperplasia Impact Index | Urinary | 1 (1%) |
| DASS | | Depression, Anxiety & Stress Scale | Anxiety/Depression | 1 (1%) |
| ECOG | | ECOG Performance Status Scale | QoL | 1 (1%) |
| HFSSS | | Hot Flush Symptom Severity Score | Hormonal | 1 (1%) |
| KHQ | | Kings Health Questionnaire | Urinary & QoL | 1 (1%) |
| MAX-PC | | Memorial Anxiety Scale for Prostate Cancer | Anxiety/Depression | 1 (1%) |
| MPQ | | McGill Pain Questionnaire | Pain | 1 (1%) |
| PSQ-18 | | Patient Satisfaction Questionnaire | Satisfaction | 1 (1%) |
| QUFW94 | | Prostate Cancer Specific Questionnaire | Urinary, Sexual & Bowel | 1 (1%) |
| STAI | | State Trait Anxiety Inventory | Anxiety/Depression | 1 (1%) |
| VAS scale | | Visual Analogue Scale - Pain | Pain | 1 (1%) |
| WPAI | | Working Productivity & Activity Impairment Qnr | QoL | 1 (1%) |
| Bespoke | | Bespoke questionnaire for the study | N/A | 5 (7%) |

**Table S5b.** Details of the patient reported outcome measures (PROMs) used in articles concerning lower urinary tract symptoms (139 PROMs across 54 articles)

| **PROM name** | **Long name** | **Main symptoms of interest** | **n (%) of articles using it** |
| --- | --- | --- | --- |
| ***Articles concerning Lower Urinary Tract Symptoms*** | | | |
| I-PSS | International Prostate Symptom Score | Urinary/QoL | 50 (93%) |
| IIEF | The International Index of Erectile Function | Sexual | 22 (41%) |
| BPHII | Benign Prostatic Hyperplasia Impact Index | Urinary | 10 (19%) |
| Diary | Bladder diary (e.g. TUFS) | Urinary | 10 (19%) |
| OAB | OverActive Bladder Questionnaire | Urinary | 9 (17%) |
| MSHQ | Male Sexual Health Questionnaire | Sexual | 6 (11%) |
| ICIQ (e.g. ICIQmluts or ICS) | The International Consultation on Incontinence Questionnaire | Urinary/Sexual/QoL/Satisfaction with treatment | 5 (9%) |
| PGI-I | Patient Global Impression of Improvement | Symptom improvement | 5 (9%) |
| SF-36/SF-12 | 36 Item/12 Item Short Form Survey | QoL | 4 (7%) |
| EuroQol (e.g. EQ-5D-5L) | EuroQol Research Foundation | QoL | 2 (4%) |
| PPBC | Patient Perception of Bladder Condition | Urinary | 2 (4%) |
| CPSI | Chronic Prostatitis Symptom Index | Urinary/Pain/QoL | 1 (2%) |
| DAN-PSS | Danish Prostate Symptom Score | Urinary/Sexual | 1 (2%) |
| HPDMQ | Hallyn Post-Micturition Dribble Questionnaire | Urinary | 1 (2%) |
| IES-R | Revised Impact of Event Scale | Stress Disorders | 1 (2%) |
| MSF | Male Sexual Function Questionnaire | Sexual | 1 (2%) |
| Nocturia QoL | Nocturia Quality of Life | Urinary/QoL | 1 (2%) |
| PEDT | Premature Ejaculation Diagnostic Tool | Sexual | 1 (2%) |
| PPSM | Patient Perception of Study Medication | Treatment satisfaction | 1 (2%) |
| PPST | Patient Perception of Study Treatment | Treatment satisfaction | 1 (2%) |
| SEP | Sexual Encounter Profile | Sexual | 1 (2%) |
| TSS-BPH | Treatment Satisfaction Scale - Benign Prostatic Hyperplasia | Treatment Satisfaction | 1 (2%) |
| UPS | Urgency Perception Scale | Urinary | 1 (2%) |
| VAS scale | Visual Analogue Scale | Urinary | 1 (2%) |
| WPAI | Work Productivity and Activity Impairment | QoL | 1 (2%) |

**Table S6a.** Number of articles* where specific PROMs were used in conjunction, in those concerning prostate cancer

|  | FACT/  FACIT | EORTC | BPI | EPIC | SF12  /36 | EuroQoL | IPSS | Diary | ICIQ | IIEF | HADS |
| --- | --- | --- | --- | --- | --- | --- | --- | --- | --- | --- | --- |
| FACT/  FACIT |  | 5 | 13 | 2 | 2 | 10 | 0 | 0 | 0 | 0 | 0 |
| EORTC | 5 |  | 3 | 1 | 2 | 2 | 0 | 0 | 1 | 0 | 1 |
| BPI | 13 | 3 |  | 0 | 0 | 7 | 0 | 0 | 0 | 0 | 0 |
| EPIC | 2 | 1 | 0 |  | 5 | 0 | 3 | 1 | 2 | 4 | 3 |
| SF12/36 | 2 | 2 | 0 | 5 |  | 0 | 2 | 0 | 1 | 2 | 3 |
| EuroQoL | 10 | 2 | 7 | 0 | 0 |  | 0 | 0 | 0 | 0 | 0 |
| IPSS | 0 | 0 | 0 | 3 | 2 | 0 |  | 1 | 1 | 2 | 1 |
| Diary | 0 | 0 | 0 | 1 | 0 | 0 | 1 |  | 0 | 1 | 0 |
| ICIQ | 0 | 1 | 0 | 2 | 1 | 0 | 1 | 0 |  | 0 | 1 |
| IIEF | 0 | 0 | 0 | 4 | 2 | 0 | 2 | 1 | 0 |  | 2 |
| HADS | 0 | 1 | 0 | 3 | 3 | 0 | 1 | 0 | 1 | 2 |  |

**Footnote**: *excludes measures used in ≤2 articles. Articles may be included more than one cell if they used more than 2 PROMs. Diary = Any bladder diary (e.g. TUFS), EORTC = European Organisation for Research and Treatment of Cancer, EPIC = Expanded Prostate Cancer Index, FACT/FACIT = Functional Assessment of Cancer Therapy/Chronic Illness Therapy, HADS = Hospital Anxiety and Depression Scale, ICIQ = The International Consultation on Incontinence Questionnaire, IIEF = The International Index of Erectile Function, IPSS = International Prostate Symptom Score, PROM = Patient Reported Outcome Measures, SF-12/-36 = 12 or 36 Item Short Form Survey

**Table S6b.** Number of articles* where specific PROMs were used in conjunction, in those concerning lower urinary tract symptoms

|  | IPSS | IIEF | BPHII | Diary | OAB | MSHQ | ICIQ | PGI-I | SF12/36 |
| --- | --- | --- | --- | --- | --- | --- | --- | --- | --- |
| IPSS |  | 20 | 10 | 9 | 9 | 6 | 5 | 5 | 3 |
| IIEF | 20 |  | 5 | 1 | 3 | 5 | 4 | 3 | 2 |
| BPHII | 10 | 15 |  | 0 | 1 | 4 | 1 | 2 | 1 |
| Diary | 9 | 1 | 0 |  | 4 | 0 | 1 | 2 | 0 |
| OAB | 9 | 3 | 1 | 4 |  | 1 | 3 | 2 | 2 |
| MSHQ | 6 | 5 | 4 | 0 | 1 |  | 1 | 0 | 2 |
| ICIQ | 5 | 4 | 1 | 1 | 3 | 1 |  | 0 | 0 |
| PGI-I | 5 | 3 | 2 | 2 | 2 | 0 | 0 |  | 0 |
| SF12/36 | 3 | 2 | 1 | 0 | 2 | 2 | 0 | 0 |  |

**Footnote**: *excludes measures used in ≤2 articles. Articles may be included more than one cell if they used more than 2 PROMs. BPHII = Benign Prostatic Hyperplasia Impact Index, BPI = Brief Pain Inventory, Diary = Any bladder diary (e.g. TUFS), ICIQ = The International Consultation on Incontinence Questionnaire, IIEF = The International Index of Erectile Function, IPSS = International Prostate Symptom Score, LUTS = Lower Urinary Tract Symptoms, MSHQ = Male Sexual Health Questionnaire, OAB = Overactive Bladder Assessment Tool, PGI-I = Patient Global Impression of Improvement, PROM = Patient Reported Outcome Measures, SF-12/-36 = 12 or 36 Item Short Form Survey
