# Supplementary material for: Patient‐reported outcome measures in prostate research: a scoping review
Source: BJU Int. 2025 Oct 10;137(2):241–50. doi: 10.1111/bju.70022 (PMC12789852; doi:10.1111/bju.70022)
Supplement: Supplementary file 3 — Appendix S3. Included articles. [file BJU-137-241-s004.docx]

**Appendix 3. Included articles**

**Table S7.** Articles included in the full text review (n=121)

| **Article title** | **Year** | **Journal** | **Authors** | **PubMed ID** |
| --- | --- | --- | --- | --- |
| Comparison of prostatic artery embolisation (PAE) versus transurethral resection of the prostate (TURP) for benign prostatic hyperplasia: randomised, open label, non-inferiority trial. | 2018 | BMJ (Clinical research ed.) | Abt D et al. | 29921613 |
| Health-related quality of life after apalutamide treatment in patients with metastatic castration-sensitive prostate cancer (TITAN): a randomised, placebo-controlled, phase 3 study. | 2019 | The Lancet. Oncology | Agarwal N et al. | 31578173 |
| Effects of bipolar and monopolar transurethral resection of the prostate on urinary and erectile function: a prospective randomized comparative study. | 2013 | BJU international | Akman T et al. | 22672229 |
| ARCHES: A Randomized, Phase III Study of Androgen Deprivation Therapy With Enzalutamide or Placebo in Men With Metastatic Hormone-Sensitive Prostate Cancer. | 2019 | Journal of clinical oncology | Armstrong AJ et al. | 31329516 |
| Androgen deprivation therapy for volume reduction, lower urinary tract symptom relief and quality of life improvement in patients with prostate cancer: degarelix vs goserelin plus bicalutamide. | 2012 | BJU international | Axcrona K et al. | 22500884 |
| A European multicenter randomized noninferiority trial comparing 180 W GreenLight XPS laser vaporization and transurethral resection of the prostate for the treatment of benign prostatic obstruction: 12-month results of the GOLIATH study. | 2015 | The Journal of urology | Bachmann A et al. | 25219699 |
| 180-W XPS GreenLight laser vaporisation versus transurethral resection of the prostate for the treatment of benign prostatic obstruction: 6-month safety and efficacy results of a European Multicentre Randomised Trial--the GOLIATH study. | 2014 | European urology | Bachmann A et al. | 24331152 |
| Impact of cabazitaxel on 2-year survival and palliation of tumour-related pain in men with metastatic castration-resistant prostate cancer treated in the TROPIC trial. | 2013 | Annals of oncology | Bahl A et al. | 23723295 |
| A Parallel Randomized Clinical Trial Examining the Return of Urinary Continence after Robot-Assisted Radical Prostatectomy with or without a Small Intestinal Submucosa Bladder Neck Sling. | 2016 | The Journal of urology | Bahler CD et al. | 26784645 |
| Abiraterone acetate plus prednisone versus prednisone alone in chemotherapy-naive men with metastatic castration-resistant prostate cancer: patient-reported outcome results of a randomised phase 3 trial. | 2013 | The Lancet. Oncology | Basch E et al. | 24075621 |
| A randomized, double-blind, solifenacin succinate versus placebo control, phase 4, multicenter study evaluating urinary continence after robotic assisted radical prostatectomy. | 2015 | The Journal of urology | Bianco FJ et al. | 25281778 |
| Long-term distress after radical prostatectomy versus watchful waiting in prostate cancer: a longitudinal study from the Scandinavian Prostate Cancer Group-4 randomized clinical trial. | 2013 | European urology | Bill-Axelson A et al. | 23465517 |
| Patient-reported Quality of Life in Patients with Primary Metastatic Prostate Cancer Treated with Androgen Deprivation Therapy with and Without Concurrent Radiation Therapy to the Prostate in a Prospective Randomised Clinical Trial; Data from the HORRAD Trial. | 2021 | European urology | Boevé L et al. | 32978014 |
| Intensity-modulated fractionated radiotherapy versus stereotactic body radiotherapy for prostate cancer (PACE-B): acute toxicity findings from an international, randomised, open-label, phase 3, non-inferiority trial. | 2019 | The Lancet. Oncology | Brand DH et al. | 31540791 |
| Impact of Radiotherapy When Added to Androgen-Deprivation Therapy for Locally Advanced Prostate Cancer: Long-Term Quality-of-Life Outcomes From the NCIC CTG PR3/MRC PR07 Randomized Trial. | 2015 | Journal of clinical oncology | Brundage M et al. | 26014295 |
| Locally advanced and metastatic prostate cancer treated with intermittent androgen monotherapy or maximal androgen blockade: results from a randomised phase 3 study by the South European Uroncological Group. | 2014 | European urology | Calais da Silva F et al. | 23582949 |
| Efficacy and safety of the coadministration of tadalafil once daily with finasteride for 6 months in men with lower urinary tract symptoms and prostatic enlargement secondary to benign prostatic hyperplasia. | 2014 | The Journal of urology | Casabé A et al. | 24096118 |
| Impact of enzalutamide on quality of life in men with metastatic castration-resistant prostate cancer after chemotherapy: additional analyses from the AFFIRM randomized clinical trial. | 2015 | Annals of oncology | Cella D et al. | 25361992 |
| Silodosin therapy for lower urinary tract symptoms in men with suspected benign prostatic hyperplasia: results of an international, randomized, double-blind, placebo- and active-controlled clinical trial performed in Europe. | 2011 | European urology | Chapple CR et al. | 21109344 |
| Plasmakinetic enucleation of the prostate compared with open prostatectomy for prostates larger than 100 grams: a randomized noninferiority controlled trial with long-term results at 6 years. | 2014 | European urology | Chen S et al. | 24502959 |
| A prospective, randomized clinical trial comparing plasmakinetic resection of the prostate with holmium laser enucleation of the prostate based on a 2-year followup. | 2013 | The Journal of urology | Chen YB et al. | 23174256 |
| Patient-reported outcomes following abiraterone acetate plus prednisone added to androgen deprivation therapy in patients with newly diagnosed metastatic castration-naive prostate cancer (LATITUDE): an international, randomised phase 3 trial. | 2018 | The Lancet. Oncology | Chi KN et al. | 29326030 |
| Safety and efficacy of once daily administration of 50 mg mirodenafil in patients with erectile dysfunction: a multicenter, double-blind, placebo controlled trial. | 2013 | The Journal of urology | Chung JH et al. | 23017527 |
| Effects of poloxamer-based thermo-sensitive sol-gel agent on urethral stricture after transurethral resection of the prostate for benign prostatic hyperplasia: a multicentre, single-blinded, randomised controlled trial. | 2020 | BJU international | Chung JH et al. | 31444917 |
| Robot-assisted laparoscopic prostatectomy versus open radical retropubic prostatectomy: 24-month outcomes from a randomised controlled study. | 2018 | The Lancet. Oncology | Coughlin GD et al. | 30017351 |
| Intermittent androgen suppression for rising PSA level after radiotherapy. | 2012 | The New England journal of medicine | Crook JM et al. | 22931259 |
| A Pragmatic Randomized Controlled Trial Examining the Impact of the Retzius-sparing Approach on Early Urinary Continence Recovery After Robot-assisted Radical Prostatectomy. | 2017 | European urology | Dalela D et al. | 28483330 |
| Conventional versus hypofractionated high-dose intensity-modulated radiotherapy for prostate cancer: 5-year outcomes of the randomised, non-inferiority, phase 3 CHHiP trial. | 2016 | The Lancet. Oncology | Dearnaley D et al. | 27339115 |
| The Oral Gonadotropin-releasing Hormone Receptor Antagonist Relugolix as Neoadjuvant/Adjuvant Androgen Deprivation Therapy to External Beam Radiotherapy in Patients with Localised Intermediate-risk Prostate Cancer: A Randomised, Open-label, Parallel-group Phase 2 Trial. | 2020 | European urology | Dearnaley DP et al. | 32273183 |
| Quality of life in men with locally advanced prostate cancer treated with leuprorelin and radiotherapy with or without zoledronic acid (TROG 03.04 RADAR): secondary endpoints from a randomised phase 3 factorial trial. | 2012 | The Lancet. Oncology | Denham JW et al. | 23151431 |
| Urodynamic effects of once daily tadalafil in men with lower urinary tract symptoms secondary to clinical benign prostatic hyperplasia: a randomized, placebo controlled 12-week clinical trial. | 2013 | The Journal of urology | Dmochowski R et al. | 23234619 |
| Patient-Reported Outcomes after Monitoring, Surgery, or Radiotherapy for Prostate Cancer. | 2016 | The New England journal of medicine | Donovan JL et al. | 27626365 |
| Long-term safety and efficacy of single-tablet combinations of solifenacin and tamsulosin oral controlled absorption system in men with storage and voiding lower urinary tract symptoms: results from the NEPTUNE Study and NEPTUNE II open-label extension. | 2015 | European urology | Drake MJ et al. | 25070148 |
| Diagnostic Assessment of Lower Urinary Tract Symptoms in Men Considering Prostate Surgery: A Noninferiority Randomised Controlled Trial of Urodynamics in 26 Hospitals. | 2020 | European urology | Drake MJ et al. | 32616406 |
| Responder and health-related quality of life analyses in men with lower urinary tract symptoms treated with a fixed-dose combination of solifenacin and tamsulosin oral-controlled absorption system: results from the NEPTUNE study. | 2016 | BJU international | Drake MJ et al. | 25907003 |
| Health-related quality of life for immediate versus delayed androgen-deprivation therapy in patients with asymptomatic, non-curable prostate cancer (TROG 03.06 and VCOG PR 01-03 [TOAD]): a randomised, multicentre, non-blinded, phase 3 trial. | 2017 | The Lancet. Oncology | Duchesne GM et al. | 28760403 |
| Phase III Study Comparing a Reduced Dose of Cabazitaxel (20 mg/m(2)) and the Currently Approved Dose (25 mg/m(2)) in Postdocetaxel Patients With Metastatic Castration-Resistant Prostate Cancer-PROSELICA. | 2017 | Journal of clinical oncology | Eisenberger M et al. | 28809610 |
| Randomised trial of bipolar resection vs holmium laser enucleation vs Greenlight laser vapo-enucleation of the prostate for treatment of large benign prostate obstruction: 3-years outcomes. | 2020 | BJU international | Elshal AM et al. | 32633020 |
| Randomised comparison of techniques for control of the dorsal venous complex during robot-assisted laparoscopic radical prostatectomy. | 2020 | BJU international | Feng T et al. | 32521115 |
| Phase III, randomized, placebo-controlled study of docetaxel in combination with zibotentan in patients with metastatic castration-resistant prostate cancer. | 2013 | Journal of clinical oncology | Fizazi K et al. | 23569308 |
| Quality of life in patients with metastatic prostate cancer following treatment with cabazitaxel versus abiraterone or enzalutamide (CARD): an analysis of a randomised, multicentre, open-label, phase 4 study. | 2020 | The Lancet. Oncology | Fizazi K et al. | 32926841 |
| Effect of enzalutamide on time to first skeletal-related event, pain, and quality of life in men with castration-resistant prostate cancer: results from the randomised, phase 3 AFFIRM trial. | 2014 | The Lancet. Oncology | Fizazi K et al. | 25104109 |
| A randomized, double-blind, placebo-controlled phase II study of maintenance therapy with tasquinimod in patients with metastatic castration-resistant prostate cancer responsive to or stabilized during first-line docetaxel chemotherapy. | 2017 | Annals of oncology | Fizazi K et al. | 29059273 |
| Long-term effects of doxazosin, finasteride and combination therapy on quality of life in men with benign prostatic hyperplasia. | 2013 | The Journal of urology | Fwu CW et al. | 23357210 |
| Bipolar plasma enucleation of the prostate vs open prostatectomy in large benign prostatic hyperplasia cases - a medium term, prospective, randomized comparison. | 2013 | BJU international | Geavlete B et al. | 23469933 |
| Continuous vs conventional bipolar plasma vaporisation of the prostate and standard monopolar resection: a prospective, randomised comparison of a new technological advance. | 2014 | BJU international | Geavlete B et al. | 24053794 |
| Influence of preoperative and postoperative pelvic floor muscle training (PFMT) compared with postoperative PFMT on urinary incontinence after radical prostatectomy: a randomized controlled trial. | 2013 | European urology | Geraerts I et al. | 23357349 |
| Acute Toxicity and Quality of Life After Dose-Intensified Salvage Radiation Therapy for Biochemically Recurrent Prostate Cancer After Prostatectomy: First Results of the Randomized Trial SAKK 09/10. | 2015 | Journal of clinical oncology | Ghadjar P et al. | 26527774 |
| A randomized trial comparing bipolar transurethral vaporization of the prostate with GreenLight laser (xps-180watt) photoselective vaporization of the prostate for treatment of small to moderate benign prostatic obstruction: outcomes after 2 years. | 2020 | BJU international | Ghobrial FK et al. | 31621175 |
| Quality-of-life outcomes from the Prostate Adenocarcinoma: TransCutaneous Hormones (PATCH) trial evaluating luteinising hormone-releasing hormone agonists versus transdermal oestradiol for androgen suppression in advanced prostate cancer. | 2017 | BJU international | Gilbert DC et al. | 27753182 |
| Functional Outcomes Following Nerve Sparing Prostatectomy Augmented with Seminal Vesicle Sparing Compared to Standard Nerve Sparing Prostatectomy: Results from a Randomized Controlled Trial. | 2017 | The Journal of urology | Gilbert SM et al. | 28392393 |
| WATER: A Double-Blind, Randomized, Controlled Trial of Aquablation(®) vs Transurethral Resection of the Prostate in Benign Prostatic Hyperplasia. | 2018 | The Journal of urology | Gilling P et al. | 29360529 |
| Thulium laser transurethral vaporesection of the prostate versus transurethral resection of the prostate for men with lower urinary tract symptoms or urinary retention (UNBLOCS): a randomised controlled trial. | 2020 | Lancet (London, England) | Hashim H et al. | 32622397 |
| Impact of Enzalutamide Compared with Bicalutamide on Quality of Life in Men with Metastatic Castration-resistant Prostate Cancer: Additional Analyses from the TERRAIN Randomised Clinical Trial. | 2017 | European urology | Heidenreich A et al. | 27497762 |
| Intermittent versus continuous androgen deprivation in prostate cancer. | 2013 | The New England journal of medicine | Hussain M et al. | 23550669 |
| Hot flushes in prostatic cancer patients during androgen-deprivation therapy with monthly dose of degarelix or leuprolide. | 2011 | Prostate cancer and prostatic diseases | Iversen P et al. | 21445092 |
| Patient-Centered Preference Assessment to Improve Satisfaction With Care Among Patients With Localized Prostate Cancer: A Randomized Controlled Trial. | 2019 | Journal of clinical oncology | Jayadevappa R et al. | 30860943 |
| Long-term quality-of-life outcomes after radical prostatectomy or watchful waiting: the Scandinavian Prostate Cancer Group-4 randomised trial. | 2011 | The Lancet. Oncology | Johansson E et al. | 21821474 |
| Efficacy and Safety of Mirabegron versus Placebo Add-On Therapy in Men with Overactive Bladder Symptoms Receiving Tamsulosin for Underlying Benign Prostatic Hyperplasia: A Randomized, Phase 4 Study (PLUS). | 2020 | The Journal of urology | Kaplan SA et al. | 31895002 |
| Safety and tolerability of solifenacin add-on therapy to α-blocker treated men with residual urgency and frequency. | 2013 | The Journal of urology | Kaplan SA et al. | 23234618 |
| Effects of hexanic extract of Serenoa repens (Permixon® 160 mg) on inflammation biomarkers in the treatment of lower urinary tract symptoms related to benign prostatic hyperplasia. | 2015 | The Prostate | Latil A et al. | 26306400 |
| Initial combined treatment with anticholinergics and α-blockers for men with lower urinary tract symptoms related to BPH and overactive bladder: a prospective, randomized, multi-center, double-blind, placebo-controlled study. | 2011 | Prostate cancer and prostatic diseases | Lee SH et al. | 21788967 |
| Effect of enzalutamide on health-related quality of life, pain, and skeletal-related events in asymptomatic and minimally symptomatic, chemotherapy-naive patients with metastatic castration-resistant prostate cancer (PREVAIL): results from a randomised, phase 3 trial. | 2015 | The Lancet. Oncology | Loriot Y et al. | 25888263 |
| Photoselective vaporization of the prostate with GreenLight 120-W laser compared with monopolar transurethral resection of the prostate: a multicenter randomized controlled trial. | 2012 | European urology | Lukacs B et al. | 22341632 |
| Midterm results from an international multicentre randomised controlled trial comparing bipolar with monopolar transurethral resection of the prostate. | 2013 | European urology | Mamoulakis C et al. | 23102675 |
| Results from an international multicentre double-blind randomized controlled trial on the perioperative efficacy and safety of bipolar vs monopolar transurethral resection of the prostate. | 2012 | BJU international | Mamoulakis C et al. | 21557796 |
| Comparison of Silodosin and Naftopidil for Efficacy in the Treatment of Benign Prostatic Enlargement Complicated by Overactive Bladder: A Randomized, Prospective Study (SNIPER Study). | 2017 | The Journal of urology | Matsukawa Y et al. | 27615436 |
| Minimally Invasive Prostate Convective Water Vapor Energy Ablation: A Multicenter, Randomized, Controlled Study for the Treatment of Lower Urinary Tract Symptoms Secondary to Benign Prostatic Hyperplasia. | 2016 | The Journal of urology | McVary KT et al. | 26614889 |
| A multicenter, randomized, double-blind, placebo controlled study of onabotulinumtoxinA 200 U to treat lower urinary tract symptoms in men with benign prostatic hyperplasia. | 2014 | The Journal of urology | McVary KT et al | 24508634 |
| Health-related quality-of-life findings for the prostate cancer prevention trial. | 2012 | Journal of the National Cancer Institute | Moinpour CM et al. | 22972968 |
| A randomised, placebo-controlled, multicentre, Phase 2 clinical trial to evaluate the efficacy and safety of GV1001 in patients with benign prostatic hyperplasia. | 2018 | BJU international | Moon KT et al. | 29633507 |
| Quality of Life During Treatment With Chemohormonal Therapy: Analysis of E3805 Chemohormonal Androgen Ablation Randomized Trial in Prostate Cancer. | 2018 | Journal of clinical oncology | Morgans AK et al. | 29522362 |
| Serenoa repens + selenium + lycopene vs tadalafil 5 mg for the treatment of lower urinary tract symptoms secondary to benign prostatic obstruction: a Phase IV, non-inferiority, open-label, clinical study (SPRITE study). | 2018 | BJU international | Morgia G et al. | 29569389 |
| Intermittent hormonal therapy in the treatment of metastatic prostate cancer: a randomized trial. | 2012 | BJU international | Mottet N et al. | 22502816 |
| Transurethral resection of prostate with plasmakinetic energy: 100 months results of a prospective randomized trial. | 2012 | BJU international | Muslumanoglu AY et al. | 22115409 |
| Duration of Androgen Deprivation Therapy in High-risk Prostate Cancer: A Randomized Phase III Trial. | 2018 | European urology | Nabid A et al | 29980331 |
| Tamsulosin and placebo vs tamsulosin and tadalafil in male lower urinary tract symptoms: a double-blinded, randomised controlled trial. | 2020 | BJU international | Nagasubramanian S et al. | 32012409 |
| Ten-year Mortality, Disease Progression, and Treatment-related Side Effects in Men with Localised Prostate Cancer from the ProtecT Randomised Controlled Trial According to Treatment Received. | 2020 | European urology | Neal DE et al. | 31771797 |
| A Randomized Study of Intraoperative Autologous Retropubic Urethral Sling on Urinary Control after Robotic Assisted Radical Prostatectomy. | 2017 | The Journal of urology | Nguyen HG et al. | 27693447 |
| Silodosin for men with chronic prostatitis/chronic pelvic pain syndrome: results of a phase II multicenter, double-blind, placebo controlled study. | 2011 | The Journal of urology | Nickel JC et al. | 21571345 |
| Patient-reported quality-of-life analysis of radium-223 dichloride from the phase III ALSYMPCA study. | 2016 | Annals of oncology | Nilsson S et al. | 26912557 |
| Impact of complete bladder neck preservation on urinary continence, quality of life and surgical margins after radical prostatectomy: a randomized, controlled, single blind trial. | 2013 | The Journal of urology | Nyarangi-Dix JN et al. | 23017512 |
| Treatment satisfaction with tadalafil or tamsulosin vs placebo in men with lower urinary tract symptoms (LUTS) suggestive of benign prostatic hyperplasia (BPH): results from a randomised, placebo-controlled study. | 2014 | BJU international | Oelke M et al. | 24612148 |
| Monotherapy with tadalafil or tamsulosin similarly improved lower urinary tract symptoms suggestive of benign prostatic hyperplasia in an international, randomised, parallel, placebo-controlled clinical trial. | 2012 | European urology | Oelke M et al. | 22297243 |
| Cabazitaxel Versus Docetaxel As First-Line Therapy for Patients With Metastatic Castration-Resistant Prostate Cancer: A Randomized Phase III Trial-FIRSTANA. | 2017 | Journal of clinical oncology | Oudard S et al. | 28753384 |
| Timing of radiotherapy after radical prostatectomy (RADICALS-RT): a randomised, controlled phase 3 trial. | 2020 | Lancet (London, England) | Parker CC et al. | 33002429 |
| Symptom relief and anejaculation after aquablation or transurethral resection of the prostate: subgroup analysis from a blinded randomized trial. | 2019 | BJU international | Plante M et al. | 29862630 |
| Efficacy and safety of tadalafil once daily in the treatment of men with lower urinary tract symptoms suggestive of benign prostatic hyperplasia: results of an international randomized, double-blind, placebo-controlled trial. | 2011 | European urology | Porst H et al. | 21871706 |
| Updated interim efficacy analysis and long-term safety of abiraterone acetate in metastatic castration-resistant prostate cancer patients without prior chemotherapy (COU-AA-302). | 2014 | European urology | Rathkopf DE et al. | 24647231 |
| Clinical outcomes after combined therapy with dutasteride plus tamsulosin or either monotherapy in men with benign prostatic hyperplasia (BPH) by baseline characteristics: 4-year results from the randomized, double-blind Combination of Avodart and Tamsulosin (CombAT) trial. | 2011 | BJU international | Roehrborn CG et al. | 21332630 |
| Influence of baseline variables on changes in International Prostate Symptom Score after combined therapy with dutasteride plus tamsulosin or either monotherapy in patients with benign prostatic hyperplasia and lower urinary tract symptoms: 4-year results of the CombAT study. | 2014 | BJU international | Roehrborn CG et al. | 24127818 |
| The prostatic urethral lift for the treatment of lower urinary tract symptoms associated with prostate enlargement due to benign prostatic hyperplasia: the L.I.F.T. Study. | 2013 | The Journal of urology | Roehrborn CG et al. | 23764081 |
| A prospective randomised placebo-controlled study of the impact of dutasteride/tamsulosin combination therapy on sexual function domains in sexually active men with lower urinary tract symptoms (LUTS) secondary to benign prostatic hyperplasia (BPH). | 2018 | BJU international | Roehrborn CG et al. | 29044968 |
| Efficacy and safety of a fixed-dose combination of dutasteride and tamsulosin treatment (Duodart(®) ) compared with watchful waiting with initiation of tamsulosin therapy if symptoms do not improve, both provided with lifestyle advice, in the management of treatment-naïve men with moderately symptomatic benign prostatic hyperplasia: 2-year CONDUCT study results. | 2015 | BJU international | Roehrborn CG et al. | 25565364 |
| Effect of apalutamide on health-related quality of life in patients with non-metastatic castration-resistant prostate cancer: an analysis of the SPARTAN randomised, placebo-controlled, phase 3 trial. | 2018 | The Lancet. Oncology | Saad F et al. | 30213449 |
| Advanced prostate cancer treated with intermittent or continuous androgen deprivation in the randomised FinnProstate Study VII: quality of life and adverse effects. | 2013 | European urology | Salonen AJ et al. | 22857983 |
| Increased survival with enzalutamide in prostate cancer after chemotherapy. | 2012 | The New England journal of medicine | Scher HI et al. | 22894553 |
| Intermittent Versus Continuous Androgen Deprivation Therapy in Patients with Relapsing or Locally Advanced Prostate Cancer: A Phase 3b Randomised Study (ICELAND). | 2016 | European urology | Schulman C et al. | 26520703 |
| Quartz head contact laser fiber: a novel fiber for laser ablation of the prostate using the 980 nm high power diode laser. | 2012 | The Journal of urology | Shaker HS et al. | 22177175 |
| Does Peak Urine Flow Rate Predict the Development of Incident Lower Urinary Tract Symptoms in Men with Mild to No Current Symptoms? Results from REDUCE. | 2017 | The Journal of urology | Simon RM et al. | 28428110 |
| Effect of Enzalutamide plus Androgen Deprivation Therapy on Health-related Quality of Life in Patients with Metastatic Hormone-sensitive Prostate Cancer: An Analysis of the ARCHES Randomised, Placebo-controlled, Phase 3 Study. | 2020 | European urology | Stenzl A et al. | 32336645 |
| Effect of abiraterone acetate on fatigue in patients with metastatic castration-resistant prostate cancer after docetaxel chemotherapy. | 2013 | Annals of oncology | Sternberg CN et al. | 23152362 |
| Effects of Different Exercise Modalities on Fatigue in Prostate Cancer Patients Undergoing Androgen Deprivation Therapy: A Year-long Randomised Controlled Trial. | 2017 | European urology | Taaffe DR et al. | 28249801 |
| A Multicenter Randomized Noninferiority Trial Comparing GreenLight-XPS Laser Vaporization of the Prostate and Transurethral Resection of the Prostate for the Treatment of Benign Prostatic Obstruction: Two-yr Outcomes of the GOLIATH Study. | 2016 | European urology | Thomas JA et al. | 26283011 |
| Patient-reported outcomes following enzalutamide or placebo in men with non-metastatic, castration-resistant prostate cancer (PROSPER): a multicentre, randomised, double-blind, phase 3 trial. | 2019 | The Lancet. Oncology | Tombal B et al. | 30770294 |
| Effect of dutasteride on clinical progression of benign prostatic hyperplasia in asymptomatic men with enlarged prostate: a post hoc analysis of the REDUCE study. | 2013 | BMJ (Clinical research ed.) | Toren P et al. | 23587564 |
| Randomized trial to assess the impact of venlafaxine and soy protein on hot flashes and quality of life in men with prostate cancer. | 2013 | Journal of clinical oncology | Vitolins MZ et al. | 24081940 |
| Low dose oral desmopressin for nocturnal polyuria in patients with benign prostatic hyperplasia: a double-blind, placebo controlled, randomized study. | 2011 | The Journal of urology | Wang CJ et al. | 21074790 |
| Combined androgen deprivation therapy and radiation therapy for locally advanced prostate cancer: a randomised, phase 3 trial. | 2011 | Lancet (London, England) | Warde P et al. | 22056152 |
| Efficacy and safety of low dose desmopressin orally disintegrating tablet in men with nocturia: results of a multicenter, randomized, double-blind, placebo controlled, parallel group study. | 2013 | The Journal of urology | Weiss JP et al. | 23454402 |
| Ultra-hypofractionated versus conventionally fractionated radiotherapy for prostate cancer: 5-year outcomes of the HYPO-RT-PC randomised, non-inferiority, phase 3 trial. | 2019 | Lancet (London, England) | Widmark A et al. | 31227373 |
| Hypofractionated radiotherapy versus conventionally fractionated radiotherapy for patients with intermediate-risk localised prostate cancer: 2-year patient-reported outcomes of the randomised, non-inferiority, phase 3 CHHiP trial. | 2015 | The Lancet. Oncology | Wilkins A et al. | 26522334 |
| Radical prostatectomy versus observation for localized prostate cancer. | 2012 | The New England journal of medicine | Wilt TJ et al. | 22808955 |
| Follow-up of Prostatectomy versus Observation for Early Prostate Cancer. | 2017 | The New England journal of medicine | Wilt TJ et al. | 28700844 |
| Preliminary results of a randomised controlled trial of an online psychological intervention to reduce distress in men treated for localised prostate cancer. | 2015 | European urology | Wootten AC et al. | 25454611 |
| Effect of tadalafil 5 mg on post-micturition dribble in men with lower urinary tract symptoms: a multicentre, double-blind, randomized, placebo-controlled trial. | 2019 | BJU international | Yang DY et al. | 31207030 |
| Robot-assisted laparoscopic prostatectomy versus open radical retropubic prostatectomy: early outcomes from a randomised controlled phase 3 study. | 2016 | Lancet (London, England) | Yaxley JW et al. | 27474375 |
| Non-inferiority of silodosin to tamsulosin in treating patients with lower urinary tract symptoms (LUTS) associated with benign prostatic hyperplasia (BPH). | 2011 | BJU international | Yu HJ et al. | 21592295 |
| Effects of Patient Centered Interventions on Persistent Urinary Incontinence after Prostate Cancer Treatment: A Randomized, Controlled Trial. | 2015 | The Journal of urology | Zhang AY et al. | 26231554 |
| Olaparib for Metastatic Castration-Resistant Prostate Cancer. | 2020 | The New England journal of medicine | de Bono J et al. | 32343890 |
| Combination therapy with solifenacin and tamsulosin oral controlled absorption system in a single tablet for lower urinary tract symptoms in men: efficacy and safety results from the randomised controlled NEPTUNE trial. | 2013 | European urology | van Kerrebroeck P et al. | 23932438 |
